# DOES EARLY-LIFE RACIAL DISCRIMINATION EXPLAIN A MENTAL HEALTH PARADOX AMONG BLACK ADULTS?

**DOI:** 10.1093/geroni/igz038.689

**Published:** 2019-11-08

**Authors:** Courtney S Thomas Tobin, Myles D Moody

**Affiliations:** 1 UCLA Fielding School of Public Health, Los Angeles, California, United States; 2 Department of sociology, University of Kentucky, Lexington, Kentucky, United States

## Abstract

To evaluate the impact of early life racial discrimination (ELRD) on mental health among Black adults. Data were from the Nashville Stress and Health Study (n=618). OLS regression models examined the relationship between ELRD and adult psychological distress; logistic regression estimated the probability of past-year major depressive disorder (MDD). We also assessed whether ELRD moderated the relationship between adult discrimination and mental health. Childhood (b=1.07, SE=0.51, p=0.04) and adolescent ELRD (b=1.32, SE=0.42, p=0.002) were associated with adult distress. Individuals who experienced childhood ERLD had 88% lower odds of adult MDD than individuals with no ELRD. Significant interaction analyses showed that ELRD was generally protective against adult discrimination. While ELRD importantly shapes distress and MDD among Black adults, patterns vary by outcome. Results indicate that adult distress and MDD develop through cumulative adversity processes that are further influenced by sensitive periods in the life course.

